# A comparative computational analysis of nonautonomous *Helitron *elements between maize and rice

**DOI:** 10.1186/1471-2164-9-467

**Published:** 2008-10-08

**Authors:** Michael Sweredoski, Leah DeRose-Wilson, Brandon S Gaut

**Affiliations:** 1 Institute for Genomics and Bioinformatics, U.C. Irvine, Irvine CA 92697, USA; 2 Dept. of Computer Science, U.C. Irvine, Irvine CA 92697, USA; 3 Dept. Ecology and Evolutionary Biology, U.C. Irvine, Irvine CA 92697, USA

## Abstract

**Background:**

*Helitrons *are DNA transposable elements that are proposed to replicate via a rolling circle mechanism. Non-autonomous *helitron *elements have captured gene fragments from many genes in maize (*Zea mays *ssp. *mays) *but only a handful of genes in Arabidopsis (*Arabidopsis thaliana*). This observation suggests very different histories for *helitrons *in these two species, but it is unclear which species contains helitrons that are more typical of plants.

**Results:**

We performed computational searches to identify *helitrons *in maize and rice genomic sequence data. Using 12 previously identified helitrons as a seed set, we identified 23 helitrons in maize, five of which were polymorphic among a sample of inbred lines. Our total sample of maize helitrons contained fragments of 44 captured genes. Twenty-one of 35 of these helitrons did not cluster with other elements into closely related groups, suggesting substantial diversity in the maize element complement. We identified over 552 helitrons in the *japonica *rice genome. More than 70% of these were found in a collinear location in the *indica *rice genome, and 508 clustered as a single large subfamily. The japonica rice elements contained fragments of only 11 genes, a number similar to that in Arabidopsis. Given differences in gene capture between maize and rice, we examined sequence properties that could contribute to differences in capture rates, focusing on 3' palindromes that are hypothesized to play a role in transposition termination. The free energy of folding for maize helitrons were significantly lower than those in rice, but the direction of the difference differed from our prediction.

**Conclusion:**

Maize *helitrons *are clearly unique relative to those of rice and Arabidopsis in the prevalence of gene capture, but the reasons for this difference remain elusive. Maize helitrons do not seem to be more polymorphic among individuals than those of Arabidopsis; they do not appear to be substantially older or younger than the helitrons in either species; and our analyses provided little evidence that the 3' hairpin plays a role.

## Background

Traditionally, transposable elements (TEs) have been classified as either Class I or Class II [1]. Class I, or retroelements, transpose through an RNA intermediate. This group includes both long-terminal repeat (LTR) transposons and non-long-terminal repeat retrotransposons [2]. Class II elements transpose via DNA, include inverted repeats [3], and often leave a "footprint" after excision [4-6]. The two classes of transposable elements share at least two features: first, both duplicate host sequences during integration into the host genome, and second, the 3' end of the insertion is either a duplicate of the 5' end or a poly (A) tail [1].

In 2001, Kapitonov and Jurka identified a new group of eukarotyic DNA TEs in eukaryotes that they named *helitrons *[7]. *Helitrons *are DNA elements that are proposed to move via a rolling circle replication mechanism similar to that of some prokaryotic transposable elements [8]. They are unique in that they do not duplicate host insertion sites and do not contain terminal repeats. They can also be quite large, up to 15 kb or more [9]. However, *helitrons *do have two conserved sequence features. First, they tend to insert between Adenine and Thymidine residues. Second, they have conserved ends consisting of TC on the 5' end and CTRR on the 3' end, often with a palindromic sequence of 16–20 bp near the 3' terminus. Because they do not replicate by the "cut and paste" method of most class II elements, *helitrons *were initially difficult to classify and were considered their own class. They have now been assigned as a separate subclass of class II TEs [1].

Non-autonomous *helitrons *are common in eukaryotic genomes, representing ~2–3% of genomes in species as diverse as *Arabidopsis thaliana *(Arabidopsis), *Caenorabditis elegans *and bats (*Myotis lucifugus*) [7,10,11], but they are arguably most interesting in maize (*Zea mays *ssp. *mays*), where they contribute to substantial differences in gene order and content among individuals [12,13]. For example, the *bronze *region features extensive sequence non-homologies among maize inbred lines [14], in large part due to non-autonomous *helitron *insertions. Remarkably, much of this non-homology represents expressed gene fragments that have been captured within non-autonomous elements. Gene capture appears to be both widespread and frequent. For example, just four *helitrons *were found to contain fragments that originated from at least seven genes [15]. Moreover, overgo hybridization suggests that as many as 10,000 genes or gene fragments may be unshared between inbred lines, perhaps due to *helitron *activity [16]. The presence of expressed gene fragments within *helitrons *highlights their potential to contribute to functional, as well as structural, diversity within plant genomes [9,13,15-18].

The observations in maize provide a puzzling contrast to observations in Arabidopsis. Arabidopsis contains >1000 non-autonomous *helitrons*, many of which were initially catalogued as *Basho *elements [19]. Like *helitrons *in maize, many *A. thaliana *elements contain gene fragments. Unlike maize, however, these fragments appear to represent only a small number of gene capture events [20]. This contrast between maize and Arabidopsis raises a number of important questions about the evolution of *helitrons *and their role in gene capture. First, are the small numbers of maize *helitrons *characterized thus far typical with respect to the extent of gene capture, or could the apparent differences between species be due to the small number of *helitrons *isolated from maize thus far? Second, if one examines a third experimental system, like rice (*Oryza sativa*), do maize or Arabidopsis *helitrons *appear to be more typical with respect to gene capture? Third, if there are real differences in gene capture between maize and other plant species, can one glean any clues as to the mechanistic or evolutionary factors that contribute to these differences?

## Results

### *Helitron *identification and polymorphism in maize

**Figure 1:**
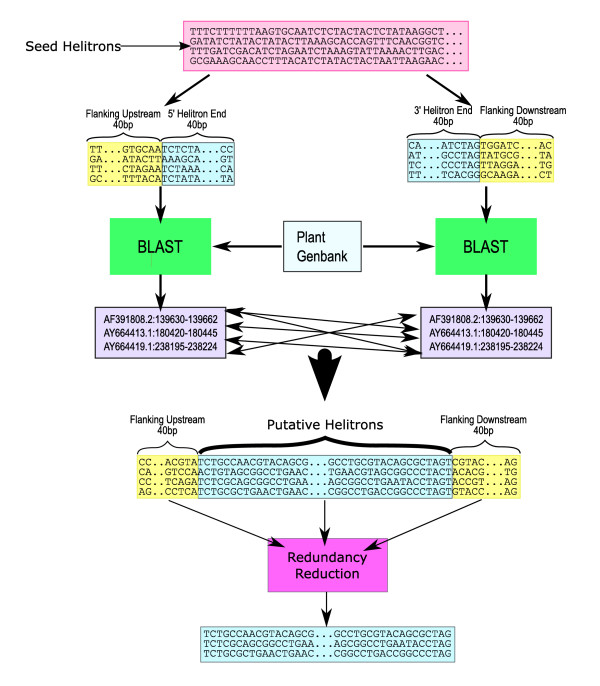
**The identification algorithm**. The flow chart diagrams the Blast-based approach used to identify *helitron *sequences initially. Approximately 40 bp were isolated from 5' and 3' ends of known *helitrons*, along with ~40 bp of flanking sequence. These ends were used as blast queries to Plant Genbank; hits to both ends that were <20,000 bp apart (represented by light blue-grey boxes with attaching arrows) were considered putative *helitrons*. These putative helitrons were screened for redundant or overlapping entries (pink box) to yield predicted *helitrons*. See Methods for details.

We used a BLAST-based approach to identify *helitrons *from maize genomic data (Figure 1). Our search began with a training set of 12 maize 'seed' elements from the literature [12,15,21,22], from which we isolated both 5' and 3' ends as BLAST queries. These queries were 80 bp in length, but generally yielded ~40 bp matches corresponding to *helitron *ends (Figure 1). This approach was applied iteratively, accruing additional BLAST queries, until no more ends were detected (see Methods). After careful consideration of redundancy in Genbank entries, we produced a curated set of 23 predicted maize *helitrons*, with a mean length of 3648 base pairs and a range from 718 bp to 7847 bp. We combined our predicted *helitrons *with the 12 query sequences to produces a total set of 35 maize *helitrons *(Table S1).

**Figure 2:**
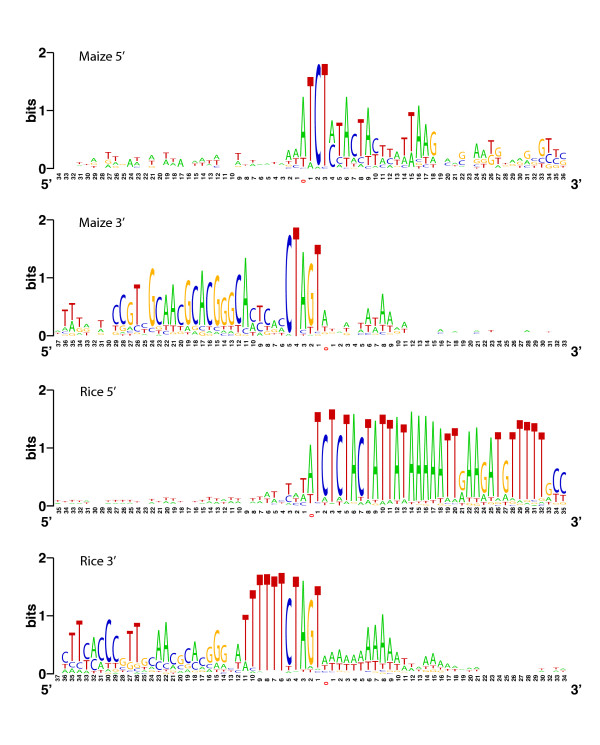
**Conserved sequence properties of *helitron *ends**. The height of the letter reflects the nucleotide conservation [36]. The x-axis represents nucleotide position; the nucleotide labeled as position '0' is the flanking nucleotide.

Before drawing conclusions from our predicted set of maize *helitrons*, it is important to examine the properties of the predicted elements. We first examined the 3' and 5' ends for features characteristic of *helitrons *(Figure 2). To date, maize *helitrons *have had 5' ends marked by insertion next to a flanking 'A' and a beginning sequence of 'TCT', followed by a 13 bp pattern without a strong consensus [18]. Du et al. [23] recently used this 18 bp pattern, including sequence ambiguities, to identify potential 5' ends of *helitrons *in maize genomic sequence. Our collection of 35 putative elements perfectly reflect the search pattern of Du et al. (2008), but also clearly indicate a lack of strong consensus nucleotides at residues 10 to 14 (Figure 2). Similarly, our sample of predicted *helitrons *reflect the recognized preference for *helitrons *to terminate in 'CTAGT', and the 3' ~30 bp motif also match the maize consensus [23,24]. Although our search began with a seed set that reflected particular properties, there is no guarantee that our BLAST strategy would return strong signals in the particular residues that have previously been defined as typical of *helitrons*. Moreover, these patterns are qualitatively indistinguishable when the 12 'seed' *helitrons *are removed from consideration (data not shown). In addition to 5' and 3' motifs, our results clearly indicate the lack of consensus nucleotides in flanking genomic sequences, except for the strong (but not absolute) bias toward inserting downstream from an 'A' (Figure 2).

Because maize *helitrons *were first identified by comparisons of sequence data between maize inbred lines [14], we next assessed polymorphism among maize individuals. We attempted to survey our 23 predicted *helitrons *with a PCR assay of eight maize inbred lines (see Methods). We were unable to amplify single bands consistently for eight of the predicted *helitrons*, and so could not unambiguously assess polymorphism for these presumptive *helitrons*. Another eight were fixed in the panel of eight maize inbred lines, and polymorphism could not be verified in a broader germplasm set, either due to a lack of polymorphism or poor amplification properties. However, five *helitrons *were polymorphic in the sample of inbred lines. Thus, 5 of our 23 predicted *helitrons *(21%) were confirmed to be polymorphic.

We next used the 5' and 3' ends from our panel of 35 putative maize *helitrons *to build profile HMM models (see Methods) and used the models to scan all maize genomic sequences in Genbank. We found that the recall rate, which is a measure of sensitivity, was very low (data not shown), indicating that the HMM could not effectively model the 5' and 3' maize motifs. The failure of the HMM approach may indicate that maize harbors a broad diversity of *helitron *subfamilies with widely variable 3' and 5' ends (see Discussion).

### *Helitron *identification and polymorphism in rice

While conducting our Blast-based search for maize *helitrons *in Genbank, we also discovered 23 putative *helitrons *in genomic rice sequences. Mirroring our approach for maize, we used the 5' and 3' ends from this panel of putative rice *helitrons *to build profile HMMs and used the models to scan two separate sources: genomic sequences of *indica *and *japonica *rice. In contrast to the HMM application to maize, the rice HMMs achieved a much higher recall rate, suggesting that rice *helitrons *may share extensive sequence similarity. The HMM models identified 552 putative *helitron *elements in the *japonica *rice genome (Table S2), and 604 elements in the *indica *rice genome. To determine whether we would expect to identify 552 elements based on chance alone, we constructed randomized rice genomes with the same length and compositional properties of *japonica *rice (see Methods) and reapplied the HMM. In five randomized genomes, the HMM failed to identify a single *helitron*, thus illustrating that our findings are not expected due to random hits to a genomic sequence of similar length and composition.

Because the *helitron *components from the rice genomes were similar and because the *japonica *genome is better annotated, we focused on the set of *japonica *elements for description. The mean length of predicted *japonica helitrons *was 441 bp, with 97% of these under 1.0 kb in length and at least two of the longest (> 3 kb) containing internal retrotransposons. None of the putative *helitrons *appear to be autonomous, based both on length and on BLASTn comparisons to putative helicase genes (data not shown). We examined the 5' and 3' ends of predicted rice *helitrons*, just as we examined them in maize (Figure 2). The rice *helitrons *generally yield stronger consensus motifs, with nucleotide biases extending for ~30 bp on the 5' end and 11 bp on the 3' end. Nonetheless, there are shared features of the motifs between maize and rice, particularly the 5' 'TCT' and the 3' 'CTAGT'. We also examined the regions flanking rice *helitrons*. Like maize, the *helitrons *exhibited a preference for a 5' flanking 'A' nucleotide. The 3' flanking sequences were markedly A+T rich, which is also a preference in maize but less pronounced (Figure 2).

**Figure 3:**
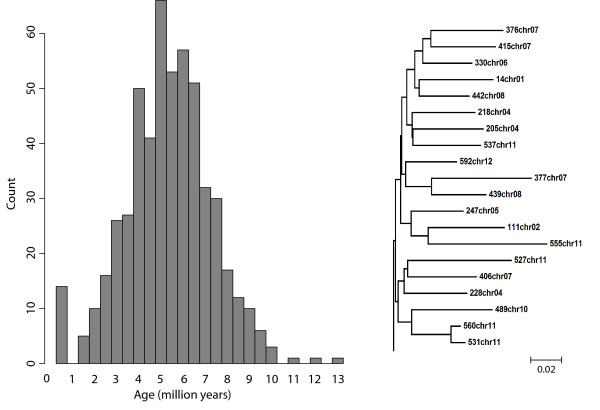
**Estimated insertion times of rice *helitrons***. The histogram of rice insertion times (*left*), which is based on terminal branch lengths (TBLs) from the phylogeny of the 508 elements in the largest rice subfamily, suggests that most *helitrons *in rice do not result from recent insertion events. A clade of the phylogeny is shown (*right*) to illustrate the general property that most TBLs are long relative to interior branches.

The strong motif pattern in rice terminal regions suggested that we had uncovered a well-conserved group of *helitrons*. To pursue this suspicion, we separated the putative *helitrons *into single-linkage clusters, yielding one particularly large cluster of 508 elements for which we constructed a neighbor-joining tree (Figure 3). With this tree, we were able to infer approximate times of element insertion. Using the terminal branch lengths (TBLs) in the phylogeny as a proxy for time, and assuming a molecular clock with a nucleotide substitution rate of 1.3 × 10^-8 ^substitutions per site per year [25], we estimate the mean insertion time for elements is ~5.1 million years ago. One interesting feature of this analysis was that there were very few short TBLs (Figure 3), which suggests a lack of recent *helitron *activity. The overall impression is that of a large family of *helitron *elements in rice that have not been particularly active in the very recent past.

We assessed polymorphism of rice *helitrons *computationally by comparing their locations between the *japonica *and *indica *genomes. To first determine orthology between *helitrons*, we compared 100 bp flanking each side of each *helitron *between genomes (see Methods). Assuming no genomic rearrangements between *japonica *and *indica*, orthologous *helitrons *should not only share flanking regions but also be in the same chromosome, on the same strand and between the same bordering flanking regions. Given this definition of orthology and a subsequent list of possible orthologous pairings, we used the maximal non-crossing matching algorithm [26] to find the one-to-one mapping with the largest cardinality. Using this approach, we estimate that 404 of the 552 *japonica *(73%) elements are found in the same location within the *indica *genome. For the 27% of *japonica helitrons *that were not found in the same location *indica*, it was not always clear if the *helitron *is truly polymorphic between genomes or if data were missing in one of the two genome sequences. However, some helitrons were polymorphic between *japonica *and *indica *with insertion sites at the predicted 3' and 5' boundaries (Fig. S1), as might be expected of recently inserted elements. Thus, our 27% estimate contains some true polymorphisms, but this figure is also the upper limit of *helitron *polymorphism between the *japonica *and *indica *genomes. The true value could be substantially lower.

### Characterization of captured coding sequences

We compared our sample of maize and *japonica *rice *helitrons *to three data sources – plant ESTs, plant CDSs, and plant genomic data – to identify gene capture events. The maize *helitrons *had hundreds of hits to EST data, but it was not always possible to determine whether this was due to homology to a known protein, due to transcription of portions of the *helitron*, or due to other unknown factors. We thus adopted conservative criteria to identify gene capture events: *i*) matches were only scored with length criteria (> 50 bp) at stringent cutoffs (e-value < e^-10^) and *ii*) gene capture was only noted if homology with CDS or genomic data included a clearly annotated gene or exon. Even with these conservative criteria, gene capture is clearly rampant within maize, as noted previously [14,15]. We identified 44 genes that had been captured by *helitrons *(Table 1). Many of the capture events were shared among our predicted and 'seed' *helitrons*, suggesting that our search yielded predicted elements with internal similarities to confirmed *helitrons*. Multiple exons were captured for several of the genes. As these capture events were duplicated via transposition, some portions of the capture event were lost from template copies, resulting in *helitrons *that retain different portions of the same gene capture event. For example, a 600 bp piece of the *oleosin KD18 *gene has been captured in *helitrons *10, 15, 20, and 27, but only the last 280 bp of this sequence is present in *helitrons *24 and 29, while *helitron *11 contains only the first 90 bp (Table 1).

**Table 1:** Genes captured by maize helitrons

Gene	No.*
19 kDa zein protein	5
transcriptional activator gene	3
heme oxygenase 1	3
ornithine carbamoyl transferase	1
rust resistance protein rp3-1	2
HC toxin reductase (hm1)	2
alcohol dehydrogenase 1 (adh1)	5
cell division like 1	3
carboxypeptidase	1
VADER mutant shrunken-2 (sh2) pseudogene	4
glyceraldehyde-3-phosphate dehydrogenase	8
phytoene synthase (Y1)	6
high sulfur zein	5
GapC2	7
male sterility restorer factor 2 (rf2a)	7
NOI protein	7
mop1	7
Rap2.7	9
22 kDa alpha zein	13
pullulanase-type starch debranching enzyme	1
Maize oleosin KD18	7
putative ribosomal protein S22 h.	1
protein phosphotase 2C PP2C	1
glucosyl transferase ribosomal protein	1
epsilon protein kinase	2
P450 monooxygenase CYP71C3v2 gene	1
tga1	1
SU1 isoamylase (sugary1)	1
Zm38	1
aberrant pollen transmission 1 (apt1)	1
cytochrome b/f complex	1
rp S8 mRNA	3
aminoadipic semialdehyde synthase	1
transcription initiation factor tfiid	2
protease S28 pro-X carboxypeptidase	1
proteasome regulatory subunits	1
ribophorin	1
ethylene-responsive factor-like protein 1 (ERF1)	1
fertilization-independent endosperm protein 1	1
lysine ketoglutarate reductase/saccharopine dehydrogenase	1
mus1	1
S1 protein (S1)	1
helix-loop-helix type transcription factor R	1
Pl-Bh (Blotched1)	1
anthocyanin regulatory C1 (c1)	1

* Number of *helitrons *containing a fragment from that gene.

In stark contrast to maize, we found evidence for only 11 unique gene capture events in the 552 *japonica *rice helitrons. Portions of these 11 genes are found in 92% (508 of 552) of the predicted elements. Six of the 11 genes are distributed widely, in that portions of each of these genes can be detected in ~200 elements (Table 2). It also is not uncommon for a *helitrons *to contain genic fragments that originated from more than one of the eleven genes – i.e., 61% (337 of the 552) of the predicted elements house genic fragments that originated from two genes. For example, portions of both the dextrinase gene and the ribosomal S12 gene are found in 121 elements. Overall, these data paint a picture of rice *helitrons *in which gene capture is infrequent but individual capture events are distributed by transposition (and perhaps recombination between elements) throughout the genome. In this respect, our sample of non-autonomous rice *helitrons *are more similar to those in Arabidopsis than those in maize. Only five gene capture events were identified in 565 Arabidopsis *helitrons*, but they too were distributed in many copies throughout the genome [20].

**Table 2:** Genes captured by rice helitrons

Gene	No.*
Os02g38690.1 protein phosphatase 2C	1
Os01g57120.1 SWIM zinc finger	1
Os06g17910.1 NBS-LRR disease resistance protein	1
Os12g42820.1 SWITCH1 splice variant S	1
Os10g01680.1 hypersensitivity-related gene	1
Os04g08270.1 limit dextrinase	215
Os10g20990.1 ribosomal protein S12	221
F-box domain containing protein	221
cytochrome P450	229
Os06g35990.1 hypothetical protein	160
Os03g43130.1hypothetical protein	228

* Number of *helitrons *containing a fragment from that gene.

### Comparative analysis of 3' palindrome folding properties

Maize *helitrons *contain more vestiges of genes than either rice or Arabidopsis *helitrons; *we postulated that this difference could be mechanistic and perhaps reflected by sequence characteristics among species. More specifically, 3' palindromes of *helitrons *are hypothesized to play a role in replication termination [10]. If this is true, we predicted that rice would have stronger 3' palindromes, facilitating less "leaky" termination, commensurately less incorporation of flanking nucleotides during replication, and therefore less gene capture. We tested this hypothesis by isolating 50 bp of the 3' of each of our *helitrons *in maize and *japonica *rice and submitting them to UNAfold analysis to estimate the average free energy of folding (d$\bar{\text{G}}$). Contrary to our prediction, the 552 predicted *japonica *rice *helitrons *have d$\bar{\text{G}}$ values significantly higher than that of our collection of maize *helitrons*, with d$\bar{\text{G}}$ = -3.50 (SD = 1.82) and d$\bar{\text{G}}$ = -5.21 (SD = 2.84) for rice and maize, respectively (t-test, p < 0.001). The difference remains significant whether one compares polymorphic *helitrons *between maize and rice (d$\bar{\text{G}}$_maize _= -5.51; d$\bar{\text{G}}$_rice _= -3.44; p < 0.001) or fixed *helitrons *between maize and rice (d$\bar{\text{G}}$_maize _= -4.94; d$\bar{\text{G}}$_rice _= -3.53; p < 0.001). In contrast there is no significant difference in d$\bar{\text{G}}$ between polymorphic and fixed *helitrons *within either species (data not shown). Thus, there are differences in 3' folding characteristics between maize and rice, but the direction of the difference was contrary to our prediction in that the rice elements have weaker 3' palindromes on average.

## Discussion

Non-autonomous helitrons can be difficult to identify both because their internal sequences are not well conserved and because they lack the obvious features of some class I and class II elements. We pursued two approaches to identify *helitrons *in maize, both of which were based on the ~40 bp 3' and 5' elements ends. An iterative, BLAST-based method yielded a non-redundant set of 23 predicted maize *helitrons*. Our predicted set of maize *helitrons *is smaller than that of Du et al. [23], for two reasons. First, we may have searched an earlier version of Genbank (the Genbank version searched by Du et al. [23] was unspecified). Second, Du et al. [23] used perl regular expression searches based on 18 and 30 bp queries for the 5' and 3' ends, respectively, which is shorter than many of our BLAST hits and may not be sufficient for appropriate identification of *helitrons *[27]. Nonetheless, 18 bp and 30 bp queries do appear to adequately cover the length of conserved 5' and 3' motifs (Figure 2).

We used our set of maize (12 'seed' *helitrons *and 23 'predicted' *helitrons*) and similarly identified rice *helitrons *to construct profile HMMs for each terminal region and each species. The rice HMMs ranked the rice training sequences near the top of the list of all potential genome hits and identified > 500 elements in each of the *japonica *and *indica *genomes, representing the first genome-wide description of putative *helitrons *in rice. Five features of this sequence collection suggest they represent bona fide rice *helitrons*: *i*) the end sequences feature known hallmarks of *helitron *3' and 5' ends (Fig. 2); *ii*) the 3' and 5' ends are found much more often than expected in random sequences; *iii*) some elements are polymorphic between *japonica *and *indica *(e.g., Figure S1); *iv*) the elements share internal similarities, even though internal similarity was not a search criterion; and *v*) the putative rice *helitrons *contain gene fragments, like *helitrons *in other plant species. The effectiveness of the rice HMMs may be due to the presence of a large, 508-member subfamily with similar sequence properties that provide a template for HMM convergence. In contrast, maize HMMs failed to achieve an acceptable rate of recall and did not effectively model the 5' and 3' motifs. One potential explanation for the failure of the maize HMM is the limited maize genomic data available at the time of the analysis. However we suspect, but cannot prove, that the failure of HMMs to converge reflects a broader diversity of *helitrons *in maize than rice, with perhaps more subfamilies of fewer members. This conjecture is supported by the fact that single-linkage clustering of our maize elements yielded a largest cluster of only 5 elements, with 21 of 35 elements remaining as singletons, suggesting maize *helitrons *share few extensive internal sequence characteristics.

Nonetheless, our number of predicted *helitrons *in maize is sufficient to categorize substantial differences among species. First, at an average length of 4616 bp, maize *helitrons *are longer than those of the other two species, with average lengths of 441 bp and 950 bp for rice and Arabidopsis, respectively. Second, both rice and Arabidopsis have clear subfamily structure. This study identified at least one major subfamily of non-autonomous elements in rice, with > 500 elements. Although several distinct subfamilies of non-autonomous *helitrons *have been identified in Arabidopsis [19], a subfamily > 200 members predominates the genomic landscape [20]. The lack of maize clusters with > 5 elements may be in part an effect of small sample, but nonetheless the difference in single-linkage clusters among species is suggestive that patterns of *helitron *diversity vary substantially among angiosperm species.

### *Helitron *polymorphism among individuals

It is also useful to compare polymorphism among individuals across maize, rice and Arabidopsis. The 12 'seed' *helitrons *in maize were identified by virtue of the fact that they were polymorphic among individuals, so this was not a random set and cannot be considered to provide an unbiased window into the rate of *helitron *presence/absence polymorphism. We did find, however, that five of our maize *helitrons *were polymorphic in a sample of 8 inbred lines, out of 15 predicted *helitrons *for which we were able to procure clean and repeatable PCR results. In rice, we found a maximal polymorphism rate of 27% between *japonica *and *indica*, and in *A. thaliana*, 51% of 278 non-autonomous elements were polymorphic in a sample of 47 individuals [20].

The percentage of polymorphic elements is not directly comparable among species because the percentage is an increasing function of the number of individuals assayed for polymorphism. However, one can compare diversity with Watterson's θ [28], which corrects for sample size and can be used as an estimate of per element *helitron *polymorphism among individuals. The estimate of θ for rice is 0.27, while that of maize and Arabidopsis are similar at 0.13 and 0.11, respectively. As noted above, the value for rice is undoubtedly inflated by incomplete sequence data between *japonica *and *indica *rice genomes. Indeed, long TBLs in the rice *helitron *phylogenies suggest that few element insertions have been recent (Figure 3), suggesting that few *helitrons *should be polymorphic between *japonica *and *indica*. Nonetheless, the fact that θ per element is similar between maize and Arabidopsis suggests that: *i*) our *helitron *predictions yield reasonable answers, in terms of the polymorphism levels of non-autonomous *helitrons *polymorphism in maize relative to the much better characterized genome of Arabidopsis and/or ii) maize *helitrons *may not be exceptionally polymorphic among individuals relative to *helitrons *in other angiosperm species.

### Gene capture

Perhaps the most puzzling difference among species is the extent of gene capture. The propensity for maize *helitrons *to capture gene fragments is well known [14-16,18]; our additional analyses of gene capture events only serve to corroborate these earlier observations (Table 1). In contrast, the relative dearth of gene fragments in non-autonomous *helitrons *has been characterized in *A. thaliana *and now rice (Table 2). Although gene capture is a predominant feature of maize *helitrons*, it may not be a common feature of plant *helitrons sensu lato*.

Why is gene capture common in maize, relative to Arabidopsis and rice? One obvious consideration is sampling biases. As described above, the complement of non-autonomous *helitrons *described to date in both Arabidopsis and rice are dominated by a single subfamily. It is possible that gene capture is for some reason particularly rare in these subfamilies, and that *helitrons *representing gene capture events have escaped identification by the different methods used in this study and the study of Arabidopsis [20]. Another sampling phenomenon could apply to maize, in that our sample of maize *helitrons *may be non-representative, thereby over-representing the prevalence of gene capture. Yet, this also seems unlikely because previously described, polymorphic *helitrons *also contain a number of gene fragments [9,15,17,18,21].

Another possibility for differences in gene capture, along with sampling phenomena, is time. Yet, the effects of time are difficult to predict precisely. One the one hand, if *helitrons *are older in maize, then there may have been more time for gene capture events to occur. On the other hand (and somewhat paradoxically), it might be reasonable to expect *younger *elements to contain more captured gene fragments. The reasoning here is that captured gene fragments, which can be expressed [15,18], might be particularly deleterious for genome function. If that is generally the case, non-autonomous *helitrons *with gene fragments will be preferentially removed from the genome over *helitrons *that do not harbor expressed gene fragments. That is, older elements should have fewer gene fragments.

Although the predicted relationship between time and gene capture is unclear, several features of our data suggest that time does not drive the observed differences among species. On the one hand, many (17 of 35 of our total sample) of the maize *helitrons *are new insertions, given that they are polymorphic among inbred lines. However, Arabidopsis *helitrons *have similar levels of polymorphism among individuals and thus are also recent. Arabidopsis elements have amplified within 5 mya [20], after the divergence of *A. thaliana *from its sister species *A. lyrata *[29]. Yet, despite potentially similar time dynamics, maize and Arabidopsis *helitrons *differ markedly in the number of gene capture events. Moreover, rice and Arabidopsis *helitrons *have comparable numbers of gene capture events, but *helitrons *in these two species seem to differ dramatically in terms of evolutionary time, with the rice elements providing little evidence of recent transposition events based on TBLs (Figure 3).

A third possible contributor to the difference in gene capture is differences in the fidelity of replication mechanisms. We proposed that the properties of 3' palindromes could differ between species, thinking that the species with weaker palindromes would have 'leakier' replication that resulted in the poor termination of replication and accompanying incorporation of flanking genes. We did find a difference in the free energy of folding between maize and rice, but it differed in direction from our prediction; maize *helitrons *have *stronger *3' palindromes than rice. This observation is not easy to reconcile with our prediction, but does call to question the functional significance of 3' palindromes.

The functional roll of palindromes in the termination of replication is at this point hypothetical [10]. In fact, Brunner et al [15] suggest that the 3' end of helitrons plays a role in the initiation (not the termination) of transposition. They base this argument on their observation that 3' ends appear to be more conserved than 5' ends; this observation is supported, but only nominally, by our 3' and 5' nucleotide profiles (Figure 2). If the 3' ends serve to initiate transposition, one might predict that more recent and active elements will have strong palindromes, but we detect no difference in d$\bar{\text{G}}$ between helitrons that are recent (i.e., polymorphic among individuals) and older (present in all assayed individuals). Added to this mystery, *helitrons *have been found recently without 3' palindromes [30], suggesting that the 3' hairpins may not be necessary for element function at all.

**Figure 4:**
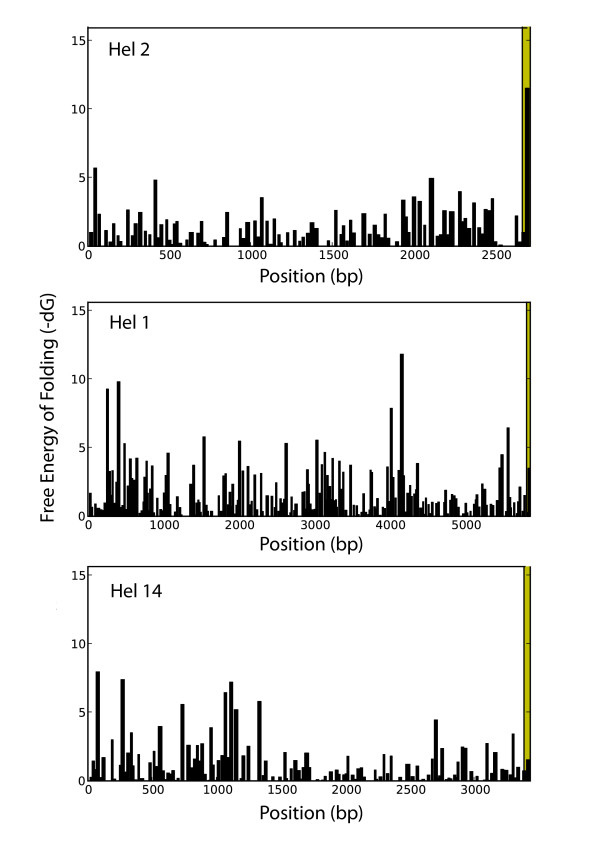
**Examples of the free energy of folding for palindromes in maize *helitrons***. *Helitrons *can be loosely categorized on the basis of the folding strength of the 3' hairpin relative to the other hairpins throughout the *helitron *sequence. The top panel shows *helitron2*, which has a strong 3' palindrome; nine of 35 maize *helitrons *exhibit this pattern. Another ten helitrons contain 3' palindromes of medium folding energy (*helitron1, center*), and 16 have 3' hairpins with a weak folding properties (*Helitr*on14, bottom). Shading indicates the region containing the most 3' palindrome.

To address bioinformatically the issue of 3' palindrome function, we asked whether the 3' palindromes of maize *helitrons *had unique properties relative to the complete *helitron *sequence. To do this, we examined all 35 maize helitrons, scanned for hairpins along their length (see Methods), estimated dG for each hairpin, and graphed dG in relation to length. Even counting liberally, only a small subset of nine of 35 *helitrons *have 3' hairpins with markedly low dG properties relative to other hairpins in the same *helitron *sequence (Figure 4). These nine *helitrons *are not biased as to whether the element is or is not demonstrably polymorphic among individuals (data not shown). Another nine *helitron *sequences had 3' hairpin structures of moderate folding strength relative to palindromes throughout the remainder of the sequence, and 16 *helitrons *had 3' hairpins without an obviously strong 3' hairpin (Figure 4). We thus find little evidence to suggest that the 3' palindromes are particularly unique within individual *helitron *sequences, suggesting that 3' palindromes either may not be necessary for *helitron *function or, at the very least, must act in concert with other signals, such as the terminal CTAG.

## Conclusion

Why do maize *helitrons *capture so many genes relative to rice and Arabidopsis? It is hard to make convincing arguments for time, enhanced polymorphism levels among individuals, or properties of 3' hairpins, and thus the answer to this question remains a mystery. One factor that we cannot address properly here is genomic defense; both rice and Arabidopsis have small genomes, and they may more effectively purge long elements (with gene capture events) to maintain genome size [20]. Another possibility is that maize has more total transposition events. The number of transposition events is the product of the potentially mobile elements (which is itself a function of the number and ratio of autonomous to non-autonomous elements) along with the transposition rate per element [31]. With more events, there is more opportunity for gene capture. There is, however, currently no data to argue for or against the idea that maize has had more transposition events in its recent evolutionary history than either maize or Arabidopsis. A third possibility is that gene capture leads almost immediately to new *helitron *subfamilies. Each new subfamily then may represent a new TE invasion event [29] that can escape genomic defenses. Hence, once gene capture begins, it can become a run-away process, but then the mystery is how it begins. Unfortunately, there is currently not enough information about *helitron *copy number in maize, the diversity of *helitrons *in maize, or the transposition rates of *helitrons *in any species to make definitive conclusions. Additional analysis of the full maize genome sequence will provide some insights, but additional information about transposition rates and population dynamics will also be critical for understanding the striking differences among *helitrons *of different angiosperm species.

## Methods

### Data sources

To start our search, we used 12 published *helitrons *to initialize our "seed set" [12,15,21,22] (Table S1). Our maize search was based on genomic data from Genbank release 149.0. Our investigation of rice included the TIGR Rice Genome Annotation, Release 5, for *japonica *rice, and data for *indica *rice genome from BGI-RIS FTP (as of 01/26/2005).

### Computational identification of *helitrons*

To identify *helitrons*, we applied two separate approaches. The first approach is represented in Figure 1. Briefly, we extracted 80 bp flanking regions of ends of 12 known maize *helitrons*, 40 bp upstream and 40 downstream from the 5' and 3' insertion sites of known *helitrons*, and used BLASTn [32] to search for paired-matches to these 80 bp terminal regions in all plant entries into Genbank release 149.0. This BLAST search generated two sets of BLAST hits, one set for 3' terminal regions and one for 5' terminal regions. Next matches for putative *helitrons *were found by looking for pairs of BLAST hits (e-value < = 1e^-5^) that were in the same GenBank entry, on the same strand, and less than 20,000 bases apart.

Many of our initial hits were actually the same region identified multiple times, both because there is redundancy in Genbank entries and also because our paired-matching criteria sometimes matched several 3' ends to one 5' end, or vice versa. Additionally some new *helitron *termini matched more than one of the 12 known *helitron *termini. To correct redundancy we examined 40 bp putative flanking regions; if both flanking regions were identical, the regions were deemed redundant. After all redundant hits were eliminated, each new putative *helitron *was hand inspected and then added to the seed set of the original 12 *helitrons *and the search was repeated. This strategy was repeated four times until no new elements were identified.

To find *helitrons *in the two available rice genomes we built a profile HMM using HMMER 1.8.5  with default values for the complete set of *helitron *terminal regions, including putative maize *helitrons *identified by the BLAST methodology described above. Using the profile HMMs, we scanned the genomes, looking for hits that were separated by at least 200 bp and no more than 20,000 bp and in the proper orientation. We then manually curated this set of putative *helitrons *to remove any remaining artifacts of the search method. To compare our results to a randomized genome, we first obtained the probabilities of observing each nucleotide given the previous two observed nucleotides. We then picked a random sequence of three nucleotides from the original sequence as our starting point and generated a random walk, given the transition probabilities, to create a new random sequence the same length as the original sequence. In this way, our randomized genomes contained not only the same base pair frequencies as the *japonica *genome but also maintained some of compositional structure (to the second order) along a sequence.

### Cataloging gene fragments

Our set of maize *helitrons *was used as a BLASTn query against three sources: *i*) all maize genomic nucleotide data available in genbank (*Zea mays *DNA sequences 12.507) *ii*) maize coding sequence (CDS) available from TIGR  and *iii*) ESTs from dbEST as of December 12, 2007 . Similarly, rice *helitrons *were used as a blastn query in a search against the rice (CDS) database from TIGR Rice Annotation Release 5.0 (January 24, 2007). An E-value cutoff of 1e^-10 ^was used for all BLAST searches, and gene fragment hits were verified manually.

### Molecular evolutionary analyses

*Helitrons *were clustered into subfamilies by single-linkage clustering, based on linkages defined by local alignments representing ≥ 60% of the shortest sequences and identities ≥ 80% over the alignment. Sequences in the largest *japonica *rice subfamily were aligned using the ClustalW multiple alignment program. This alignment was manually checked using BioEdit [33]. MEGA 3.1 [34] was used to build a neighbor-joining phylogeny using pairwise deletion, the Kimura 2-parameter substitution model [35] and 5,000 bootstrap replicates. For each species, Watterson's estimate of θ [28] was calculated on the basis of a presence/absence matrix of all *helitrons *in all individuals. Conserved nucleotides were estimated with WebLogo [36].

### Assessing polymorphism

In maize, we assessed polymorphism experimentally by performing a PCR survey in a panel of maize inbred lines. For each putative maize *helitron *three primers were designed, two flanking primers outside the *helitron *and one internal primer within the putative element. Following Hollister and Gaut (2007), two PCR amplifications were performed for each *helitron*: one reaction that flanked the putative *helitron*, and a second reaction that includes a flanking primer with a primer internal to the predicted *helitron *element. Primer sequences are available (Table S1). All primers were designed using Primer3 [37], with default conditions. Sequence from maize inbred line B73 was used to design the majority of the putative *helitron *primers; however several *helitron *primer sets were designed based on sequence information from other inbred lines such as BSS53 (Supp. Table X). All PCR utilized a 58/51 touchdown protocol with 1 min denaturing at 95C, 45 s annealing at 58C, and 1.5 min extension at 70C for 15 cycles and 10 cycles of 1 min denaturing at 95C, 45 s annealing at 51C, and 1.5 min extension at 70C followed by a 7 min elongation period at 70C. Element presence and absence was assessed in a panel of eight inbred lines, including B73 (often as a positive control for presence), Mo17, Tx601, W153, Ky21, T8, Mo24 and OH43. Additionally, some elements were assessed for presence/absence in a panel of six maize landraces and two *Zea mays *ssp. *parviglumis *individuals.

Polymorphism for rice *helitrons *was assessed computationally by comparing the *indica *and *japonica *genomes. We concatenated sequences 100 bp upstream and 100 bp downstream of the *helitrons *to find possible pairings between the two rice genomes. Blast hits of these concatenated sequences between *japonica *and *indica *were considered homologues with e-values < = 1e^-40^. The ordering of the *helitrons *within each chromosome was determined by their position in the sequence. For the *indica *genome, we also had to order the superscaffolds, which was done by aligning the superscaffolds from *indica *to the whole chromosomes in *japonica *using MUMmer [38]. Given this information, we applied the maximal non crossing matching algorithm to the ordered *helitrons*, ultimately estimating the maximum proportion of homologous *helitrons *in collinear order [26].

### Palindrome analysis

The free energy of folding (dG) of sequence fragments was estimated with UNAFold [39]. To examine the distribution of dG along complete *helitron *sequences, we first identified sequences between 4 and 20 bp that had an inverted sequence with a loop length between 1 and 10 bp and score greater than zero, when the score was calculated as the number of matches minus 2 times the number of mismatches.

## Authors' contributions

MS and LD participated in study design, carried out analyses and helped draft the manuscript. BG conceived of the study, participated in its design and coordination, and drafted the manuscript. All authors read and approved the final manuscript.
